# Childhood Trauma, Posttraumatic Stress Disorder, and Alcohol Dependence

**DOI:** 10.35946/arcr.v34.4.05

**Published:** 2012

**Authors:** Kathleen T. Brady, Sudie E. Back

**Affiliations:** **Kathleen T. Brady, M.D., Ph.D.,***is a Distinguished University Professor and director of the Clinical Neuroscience Division at the Medical University of South Carolina and the Ralph H. Johnson Veterans Affairs Medical Center, Charleston, South Carolina.*; **Sudie E. Back, Ph.D.,***is associate professor of Psychiatry and Behavioral Sciences at the Medical University of South Carolina and the Ralph H. Johnson Veterans Affairs Medical Center, Charleston, South Carolina.*

**Keywords:** Alcohol dependence, alcohol use disorders, childhood, childhood trauma, trauma-related symptoms, posttraumatic stress disorder, coping with stress or anxiety, neurobiology, biological mechanisms, treatment, pharmacological intervention, behavioral intervention, integrative psychosocial intervention, adverse child-rearing environment

## Abstract

Early-childhood trauma is strongly associated with developing mental health problems, including alcohol dependence, later in life. People with early-life trauma may use alcohol to help cope with trauma-related symptoms. This article reviews the prevalence of early-childhood trauma and its robust association with the development of alcohol use disorders and posttraumatic stress disorder. It also examines the potential biological mechanisms by which early adverse experiences can result in long-lasting changes in neurobiology underlying this vulnerability, as well as pharmacological and behavioral interventions. Recent investigations highlight the importance of assessing trauma among patients with alcohol use disorders and the positive benefits associated with the application of integrative psychosocial interventions that target both trauma-related symptoms and alcohol dependence.

Children exposed to severe adversity early in life are at increased risk of subsequently developing mental health problems, including alcohol dependence. In general, the onset of trauma precedes the onset of alcohol dependence. Although it is impossible to establish a direct causal relationship, this temporal relationship suggests a robust and positive relationship between exposure to early-life trauma and alcohol-related problems later in life. People with trauma-related symptoms and other negative consequences of early-life trauma may use alcohol to help mitigate such symptoms. People with both a positive history of early childhood trauma and co-occurring alcohol dependence have a more severe clinical profile, as well as worse treatment outcomes when compared with those with either early trauma or alcohol dependence alone. Recent investigations highlight the importance of assessing trauma among patients with alcohol use disorders and the positive benefits associated with the application of integrative psychosocial interventions that target both trauma-related symptoms and alcohol dependence. This article reviews the prevalence of early-childhood trauma and its robust association with the development of alcohol use disorders and posttraumatic stress disorder (PTSD). It also examines the potential biological mechanisms by which early adverse experiences can result in long-lasting changes in neurobiology underlying this vulnerability, as well as pharmacologic and behavioral interventions.

## Prevalence

There is little doubt that severe childhood adversity may place an individual at life-long risk for a variety of problems, including those related to mental health, physical health, employment, and legal difficulties (Putnam 2006). In a study conducted by the Centers for Disease Control and Prevention and Kaiser Permanente (Adverse Childhood Experiences [ACE] study; Felitti et al. 1998), a sample of 17,337 adults recruited from a large health maintenance organization were surveyed concerning a range of adverse events that might occur during childhood (e.g., physical or sexual abuse, incarcerated household member, emotional neglect) and adult risk behaviors, health status, and disease. The investigators found a graded relationship between the number of adverse childhood experiences (i.e., ACE score), risk behaviors during adulthood, and leading causes of morbidity and mortality in the United States, including heart disease, diabetes, liver disease, and emphysema. It is possible that these increased rates of medical conditions are not a direct result of childhood adversity but rather the result of dysfunctional and unhealthy behaviors in which many victims of childhood abuse engage.

A number of studies also report that victims of child maltreatment are more likely to have emotional difficulties and psychiatric disorders. One of the most consistent results across these studies is the finding that childhood maltreatment is associated with an increased risk for alcohol and drug use disorders (Enoch 2011). In a population-based sample of 1,411 female adult twins, self-reported childhood sexual abuse was positively associated with a number of psychiatric disorders, but the strongest associations were with alcohol and drug dependence (Kendler et al. 2000). In the ACE study, the risk of alcohol dependence increased 7.2-fold, and illicit drug use increased 4.5-fold for people with four or more ACEs (Anda et al. 2006). People with a history of childhood abuse or neglect are vulnerable to using alcohol in order to cope with stressful situations, which in turn may lead to excessive alcohol use (Schuck and Widom 2001). An investigation by Widom and colleagues (2007) demonstrates that the increased risk of excessive alcohol use among victims of childhood abuse or neglect is consistent and stable into middle adulthood (e.g., age 40). Furthermore, research has shown that alcohol-dependent patients with a history of sexual abuse are more likely than nonabused patients to relapse to alcohol use (87.5 vs. 63.3 percent) and to relapse more quickly (median time to first drink = 60 vs. 115 days) in the first year following inpatient treatment for alcohol dependence (Greenfield et al. 2002).

In addition to alcohol use disorders, childhood adversity is associated with an increased risk of PTSD (Widom 1999). Data from a number of studies over the last 20 years have emphasized the high co-occurrence of PTSD and alcohol disorders. For example, among 3,768 female twins participating in the longitudinal Missouri Adolescent Female Twin Study (MOAFTS), Sartor and colleagues (2010) found that women exposed to trauma were nearly twice as likely to develop alcohol dependence (hazard ratio 1.85), and women exposed to trauma who also had PTSD were even more likely to develop alcohol dependence (hazard ratio 3.54; significantly higher than women with trauma exposure alone) when compared with women who had not experienced trauma. Studies of samples of individuals seeking treatment for alcohol use disorders also find a high prevalence of reported childhood adversity and PTSD. In a study of men and women in treatment for addictions, 62 percent reported having been victims of childhood physical or sexual abuse (Grice et al. 1995). A review of studies of individuals seeking treatment for addictions reveals rates of PTSD as high as 50 percent or greater (Dansky et al. 1994). In the majority of cases, the development of PTSD precedes the development of the substance use disorder.

These high rates of childhood victimization in individuals with PTSD and alcohol and other substance-related problems suggests that there is a link between childhood adversity and the development of these disorders, although it is impossible to establish a direct causal relationship. However, even when studies control for demographic differences, family discord, and parental pathology, the specific relationship between childhood abuse and the development of substance use disorders holds true. Several theoretical connections have been postulated (Miller et al. 1993). Childhood victimization may lead to low self-esteem and the subsequent use of alcohol to deal with negative cognitions. It also is possible that victims of childhood abuse feel that their experiences make them “different” from other children and lead them to withdraw from healthier social circles toward fringe groups, where alcohol use is more accepted. In any case, given that victims of child abuse are more likely to develop alcohol use disorders as adults, early intervention, prevention, and training for parents are all important in interrupting this cycle of violence and alcohol problems.

## Neurobiology

Recognizing the pervasive and detrimental effects of adverse childhood experiences on quality of life and health outcomes has led to the exploration of potential biological mechanisms by which early experiences can produce long-lasting changes. Evidence from both animal and human research suggests that early stressors can lead to neurobiological changes in systems known to be involved in the pathophysiology of depression, anxiety, and substance use disorders (De Bellis et al. 1999; Heim and Nemeroff 2001). The hypothalamic–pituitary–adrenal (HPA) axis plays a critical role in the stress response and is involved in the pathophysiology of addictive disorders. Early stressors cause long-term increases in the stress response of the hormone cortisol (Plotsky and Meaney 1993) as well as decreased genetic expression of cortisol receptors and increased expression of corticotropin-releasing factor in the hypothalamus, both of which may contribute to dysregulation of the HPA axis (Ladd et al. 1996). The noradrenergic system also plays a key role in stress (Bremner 2003), and early stressors can lead to long-term decreases in α-2 noradrenergic receptors in the locus coeruleus, which may lead to loss of feedback inhibition of noradrenergic activity with associated increases in the noradrenergic stress responses (Caldji et al. 1998; Sanchez et al. 2001).

In addition to the long-lasting effects of early trauma on the stress response, a number of studies indicate that early trauma has specific effects on the neurotransmitter systems involved in the positive reinforcing effects of alcohol and drugs, particularly the brain pathway for dopamine (i.e., the mesocorticolimbic dopamine system) (Meaney et al. 2002). Higley and colleagues (1991) found that adult rhesus monkeys raised in peer groups without maternal care showed increased HPA response to stress and increased alcohol consumption during periods of stress (Higley et al. 1991). In a series of studies, Meaney and colleagues (2002) demonstrated that repeated periods of maternal separation in the early life of rats decreased dopamine transporter expression and increased dopamine responses to stress and behavioral responses to stress, cocaine, and amphetamine. These findings suggest that early-life experiences can affect the development of the mesocorticolimbic dopamine system and lead to a vulnerability to addiction in later life. Thus, in addition to effects on stress reactivity, early-life events might predispose individuals to the development of alcohol use disorders by directly influencing the reinforcing effects of alcohol. Other neurotransmitter systems involved in the pathophysiology of alcohol dependence, such as brain-derived neurotrophic factor (BDNF), serotonin, and γ-aminobutyric acid (GABA) systems also are affected by early-life trauma in ways that may influence vulnerability to the development of alcohol dependence, but the mechanistic connections in these systems are under active investigation and are not as well understood (Enoch 2011).

Not all children exposed to early-life trauma develop alcohol dependence or other significant pathology, clearly suggesting that resilience and mediating factors play a role (Enoch 2011). The genetic risk for alcohol and drug dependence involves multiple genes. Emerging evidence suggests that variation in some stress-related genes may determine the risk for psychopathology or resilience in people exposed to early-life trauma. In particular, it seems that there are important variations in the genes encoding the CRF system that can influence the development of alcohol dependence following an early-life trauma in a gene-by-environment interaction. One study of at-risk children found an interaction between a particular genetic variant coding for the CRF receptor (i.e., CRHR1) and sexual trauma in adolescents that predicted an earlier age of onset of drinking and heavy alcohol consumption (Blomeyer et al. 2008). This finding is supported by animal studies demonstrating that the CRHR1 genotype and expression interact with environmental stress to reinstate alcohol-seeking in rodents (Hansson et al. 2006), and a functional CRF promoter variant in monkeys conferred increased stress reactivity and was associated with increased alcohol consumption in animals reared under stressful conditions (Barr et al. 2009). These findings suggest that the interaction of genetic susceptibility and environmental exposure can lead to a pathologically activated CRF system, which increases the risk for the development of alcohol dependence in some people.

## Treatment

Both behavioral and pharmacological interventions are important to consider in the treatment of alcohol dependence and trauma/PTSD (Davis et al. 2006; Weiss and Kueppenbender 2006). To date, most empirical studies of behavioral or pharmacological agents have investigated the treatment of either alcohol dependence or PTSD alone.

### Psychosocial Interventions

With regard to psychosocial interventions, cognitive–behavioral therapies (CBTs) are the most widely studied and empirically valid treatments for both PTSD and alcohol use disorders. The CBTs used to treat PTSD fall into three main categories: (1) exposure-based therapies, (2) cognition-focused therapy, and (3) anxiety/stress-management therapy. Exposure-based therapies are considered the gold standard treatment for PTSD (Institute of Medicine 2008) and involve having patients confront safe, but anxiety-provoking situations (i.e., physical location where childhood abuse occurred), known as in vivo exposure; and the memory of the traumatic experience, known as imaginal exposure (Foa et al. 2006). With prolonged, repeated in vivo and imaginal exposure, the trauma-related anxiety is extinguished. Cognition-focused therapy includes cognitive therapy, which addresses the meaning that people assign to early-life trauma; and cognitive-processing therapy, which combines a narrative element of exposure therapy with efforts to identify and modify unhelpful cognitions related to the themes of safety, trust, power, esteem, and intimacy (Resick and Schnicke 1992). Finally, stress inoculation training (Meichenbaum and Novaco 1985), one of the most widely used and empirically investigated forms of anxiety management therapies, aims to provide a sense of mastery over PTSD symptoms by teaching patients a variety of coping skills. Stress inoculation training also has been incorporated into CBTs for substance use disorders and includes relaxation training, breathing retraining, thought stopping, self-instruction training, assertiveness training, cognitive restructuring, anger management, and problem solving.

Recently, integrative psychosocial interventions have been developed to address both trauma/PTSD and substance use disorders simultaneously (Back 2010). Clinicians previously believed that trauma interventions were inappropriate until after a patient had been abstinent from alcohol or drugs for a sustained period of time (e.g., 3 months). This model, known as the “sequential” model, posits that continued alcohol use impedes therapeutic efforts to address and process the trauma, and that trauma interventions commenced before sustained abstinence would result in increased risk of relapse. Contrary to these beliefs, however, recent data reported by several different investigators in the United States and Australia show that treatment outcomes of substance dependent patients who engage in integrative CBT interventions typically experience significant improvements in both conditions and that rates of relapse are not increased by the introduction of therapy for trauma (Brady et al. 2001; Hien et al. 2004; McGovern et al. 2009; Najavits 2002; Triffleman et al. 1999). Proponents of integrative treatments posit that unprocessed trauma-related memories and PTSD symptoms may, at least in part, drive alcohol use. Thus, attending to and treating the trauma-related symptoms early in the process of therapy may improve the chances of long-term recovery from alcohol (Back et al. 2006; Hien et al. 2010). Although more randomized controlled trials of integrative treatments are needed, the studies to date clearly demonstrate that for the majority of alcohol-dependent patients with trauma/PTSD, the inclusion of trauma interventions confers substantial therapeutic benefits.

## Pharmacological Interventions

There are several general issues to consider when treating co-occurring alcohol dependence and trauma/PTSD. When pharmacological agents are used, treatment should generally follow routine clinical practice for the treatment of PTSD. Regardless, relapse is common, and it is critical to consider the potential toxic interactions that may occur between the prescribed medication and alcohol. Given the high co-occurrence of alcohol and illicit drug use, potential toxic interactions between the prescribed medication and other substances of abuse must also be addressed. The pharmacological agent with the least abuse liability potential should be chosen for this population. Although benzodiazepines are effective in providing immediate relief of anxiety symptoms, they are generally not considered a first-line treatment for patients with alcohol dependence given the abuse potential of benzodiazepines. During the initial phase of treatment, when latency of onset of antidepressants is an issue, benzodiazepines may be considered as adjunctive medication. The amount of benzodiazepines prescribed to the patient should be limited, and the patient should be closely monitored for relapse or nonmedical use of benzodiazepines or other medications.

The use of pharmacological agents to specifically target alcohol dependence and PTSD is underexplored. Most studies to date, however, show promise and suggest that patients with co-occurring alcohol dependence and trauma/PTSD respond well to standard PTSD pharmacotherapies. Sertraline, a serotonin-specific reuptake inhibitor, has been investigated in patients with comorbid alcohol dependence and PTSD. The first study was a small (*n* = 9) open-label, 12-week trial, which demonstrated significant pre–post decreases in alcohol use severity (e.g., number of drinking days, number of drinks per day), as well as PTSD symptoms of re-experiencing the trauma, avoidance, and hyperarousal (Brady et al. 1995). A second study examined the efficacy of 12 weeks of sertraline compared with placebo in 94 patients with alcohol dependence and PTSD (Brady et al. 2005). The primary outcome analysis indicated no significant effect of sertraline on alcohol-related outcomes and only trend-level findings for the PTSD outcomes. The sertraline-treated group showed statistical trends for greater improvement in the experience of sudden flashbacks of the traumatic event and hyperarousal symptoms (e.g., insomnia, inability to concentrate). Follow-up cluster analyses suggested that individuals with primary PTSD, compared with primary alcohol dependence, derived more benefit from sertraline treatment as evidenced by significantly less severe alcohol use. The results suggested that patients with early-onset alcohol dependence actually had worse alcohol-related outcomes with sertraline treatment compared with placebo (Brady et al. 2005).

In another study of 254 veterans with alcohol dependence and a variety of co-occurring mood and anxiety disorders (Petrakis et al. 2005), naltrexone, disulfiram, or a combination of both was added to treatment as usual. A high percentage (42.9 percent) of the study participants had PTSD, although data analysis for specific disorders was not conducted. Alcohol-related outcomes improved significantly in patients treated with either medication alone or with combination therapy, compared with placebo, but there was no added improvement with combination therapy when compared with monotherapy. This study strongly suggests that alcohol-dependent patients with co-occurring PTSD should receive medications targeting alcohol consumption.

There is good rationale for the exploration of a number of other compounds in the treatment of co-occurring PTSD and alcohol dependence. Prazosin blocks a specific α_1_-adrenergic receptor and has shown promise in several well-controlled trials for the treatment of PTSD, particularly in decreasing PTSD-related sleep disturbance and nightmares (Raskin et al. 2007). In a preliminary study, prazosin decreased alcohol consumption in an alcohol-dependent population (Simpson et al. 2009). This inexpensive and relatively safe drug warrants investigation in the treatment of co-occurring PTSD and alcohol dependence. In addition, several anticonvulsant agents, such as topirimate, have shown promise in the treatment of alcohol dependence (Johnson et al. 2003). It is hypothesized that actions on the glutamatergic systems might be responsible for these agents’ therapeutic actions. PTSD also has been associated with glutamatergic dysregulation, and anticonvulsant agents have shown promise in small-number, open-label studies in the treatment of PTSD. This is another area in which additional investigation is warranted. More research clearly is needed to help advance the behavioral and pharmacological treatment of co-occurring trauma/PTSD and substance use disorders.

## Conclusions

Epidemiologic studies as well as studies in treatment-seeking populations converge to support the finding that early-life trauma is common in people with alcohol dependence. There are a number of potential mechanistic explanations for the connection between early-life trauma and the development of alcohol dependence. These include psychological and developmental issues that are affected by trauma, as well as neurobiological effects of early trauma that can lead to increased vulnerability to the development of alcohol and other substance use disorders. These explanatory hypotheses are not mutually exclusive. There is a growing literature on efficacious psychotherapeutic and pharmacotherapeutic treatments for individuals with co-occurring PTSD and alcohol dependence. Integrative psychosocial interventions combining efficacious interventions from the alcohol and PTSD fields have shown promise. Evidence suggests that agents targeting alcohol consumption (i.e., disulfiram, naltrexone) can be useful in patients with co-occurring PTSD and alcohol dependence, but additional investigation clearly is needed.
